# Hidden heterochromatin: Characterization in the Rodentia species *Cricetus cricetus*, *Peromyscus eremicus* (Cricetidae) and *Praomys tullbergi* (Muridae)

**DOI:** 10.1590/S1415-47572009000100009

**Published:** 2009-03-01

**Authors:** Ana Paço, Filomena Adega, Henrique Guedes-Pinto, Raquel Chaves

**Affiliations:** Institute for Biotechnology and Bioengineering, Centre of Genetics and Biotechnology, University of Trás-os-Montes and Alto Douro, Vila RealPortugal

**Keywords:** constitutive heterochromatin, *in situ restriction endonuclease digestion*, Rodentia

## Abstract

The use of *in situ* restriction endonuclease (RE) (which cleaves DNA at specific sequences) digestion has proven to be a useful technique in improving the dissection of constitutive heterochromatin (CH), and in the understanding of the CH evolution in different genomes. In the present work we describe in detail the CH of the three Rodentia species, *Cricetus cricetus*, *Peromyscus eremicus* (family Cricetidae) and *Praomys tullbergi* (family Muridae) using a panel of seven REs followed by C-banding. Comparison of the amount, distribution and molecular nature of C-positive heterochromatin revealed molecular heterogeneity in the heterochromatin of the three species. The large number of subclasses of CH identified in *Praomys tullbergi* chromosomes indicated that the karyotype of this species is the more derived when compared with the other two genomes analyzed, probably originated by a great number of complex chromosomal rearrangements. The high level of sequence heterogeneity identified in the CH of the three genomes suggests the coexistence of different satellite DNA families, or variants of these families in these genomes.

## Introduction

Constitutive heterochromatin (CH) is a ubiquitous and abundant component of eukaryotic genomes that accounts for ~30% of the genome in humans and up to 50% in the kangaroo rat (*Dipidomys ordii*) (Singer, 1982; Dimitri *et al.*, 2004, 2005; Rossi *et al.*, 2007). The similarity in the genetic and molecular properties of CH among plants and animals, led to the traditional view of this genome fraction as a “genomic wasteland” or a repository of “junk” DNA (John, 1988). Nowadays this idea is becoming obsolete; in fact, in the past two decades molecular genetics studies have implicated CH in important cellular functions, in a remarkable structural and functional basis (Dimitri *et al.*, 2004, 2005; Corradini *et al.*, 2007; Rossi *et al.*, 2007).

Constitutive heterochromatin can occur as large blocks or discrete C-positive bands in any part of a chromosome, but is most commonly found in large blocks near the centromere (Corradini *et al.*, 2007; Probst and Almouzni, 2008). Satellite DNA, the main constituent of this genomic fraction, usually occurs in the centromeric region of chromosomes (Chaves *et al.*, 2000), but is also frequently found at telomeres (Shore, 2001). The occurrence of CH at interstitial positions is much less common, although large blocks of interstitial CH have been found in the large chromosomes of some insects (John *et al.*, 1985), plants (Bauchan and Hossain, 1999) and some mammals (Santos *et al.*, 2004; Adega *et al.*, 2007; Meles *et al.*, 2008).

Although present in almost all eukaryotes, the sequence and chromosomal organization of CH is not well conserved among species. Indeed, there is strong evidence for the sharing of homologous satellite DNA sequences by closely related species (Waye and Willard, 1989; Jobse *et al.*, 1995; Lee *et al.*, 1999; Saito *et al.*, 2007), with species-specific sequences of satellite DNA occurring in almost all taxonomic groups (Slamovits and Rossi, 2002).

It seems reasonable to accept that the presence of CH facilitates the occurrence of chromosome rearrangements, as it is in accordance with several authors that consider CH as hotspots for structural chromosome rearrangements (Yunis and Yasmineh 1971; Peacock *et al.*, 1982; John, 1988; Chaves *et al.*, 2004b). Wichman *et al.* (1991) postulated that rapidly evolving families or variants of satellite DNA can promote chromosomal rearrangements via of their intragenomic movements among non-homologous chromosomes and between different chromosomal regions such as centromeres, arms and telomeres.

Sequences of CH can be easily detected by the preferential “loss” of DNA from non-C-band regions of chromosomes (Comings, 1973; Pathak and Arrighi, 1973), achieved by conventional C-banding, involving depurination and denaturation of chromosomal DNA (Arrighi and Hsu, 1971; Sumner, 1972) followed by its extraction during incubation in a saline solution (Holmquist and Dancis, 1979; Verma and Babu, 1995). Nevertheless other analytical methodologies are indispensable when a detailed molecular characterization of CH is the central issue. The use of *in situ* restriction endonuclease (RE) digestion proved to be a very useful technique in improving the dissection of CH, and in the understanding of the CH evolution in different genomes (Gosálvez *et al.*, 1997; Pieczarka *et al.*, 1998). Besides the ability of REs followed by C-banding in demonstrating the C-heterochromatin heterogeneity (Rocco *et al.*, 2002; Schmid *et al.*, 2002; Chaves *et al.*, 2004b; Adega *et al.*, 2005).

In this work, we used seven restriction endonucleases followed by C-banding to study the heterochromatin of three Rodentia species, *Cricetus cricetus*, *Peromyscus eremicus* (family Cricetidae) and *Praomys tullbergi* (family Muridae). In rodents' chromosomes, *in situ* REs digestion was only applied without sequential C-banding and only in *Microtus savii* (Galleni *et al.*, 1992), species from the genus *Reithrodontomys* (Van Den Bussche *et al.*, 1993) (family Muridae) and from the genus *Ctenomys* (family Octodontidae) (García *et al.*, 2000a, 2000b). The approach used here allowed a detailed CH characterization in terms of its location, detection of different CH subclasses, and revelation of its molecular composition.

**Figure 1:**
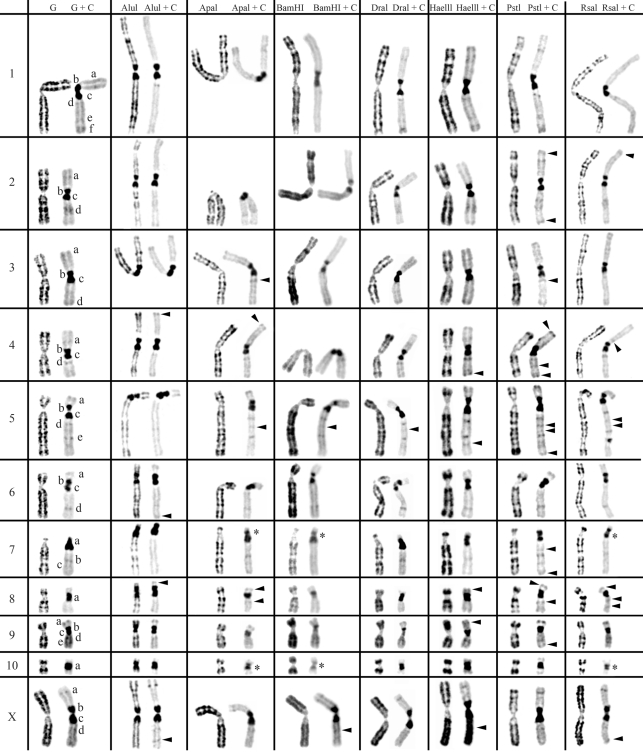
Table resume of *in situ* restriction endonuclease digestion of *Cricetus cricetus* chromosomes (2n = 22) and sequential C-banding. Control G- and C-banding of *Cricetus cricetus* chromosomes are shown on the left column. The other columns show the bands produced by the seven restriction endonucleases before and after C-banding. The letters (a-f) represent the C-bands according to their order of appearance in each chromosome. Arrowheads indicate C-positive heterochromatin bands only revealed by previous RE treatment. Asterisks indicate extra C-bands produced by the splitting of a control C- band after endonuclease digestion+C-banding.

**Figure 2 fig2:**
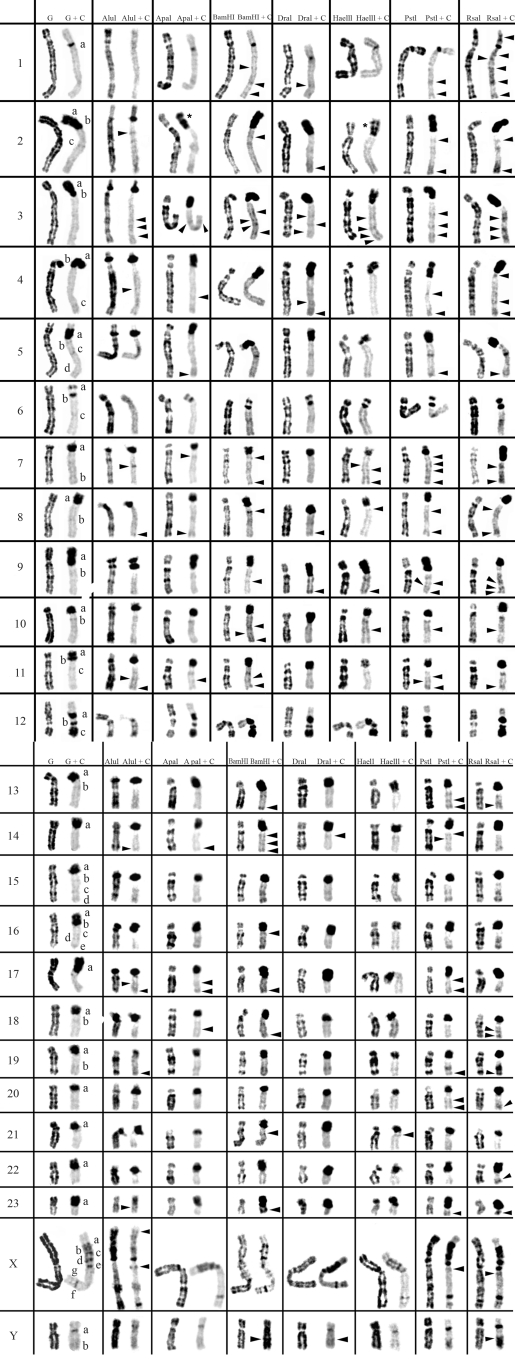
Table resume of *in situ* restriction endonuclease digestion of *Peromyscus eremicus* chromosomes (2n = 48) and sequential C-banding.  Control G- and C-banding of *Peromyscus eremicus* chromosomes are shown on the left column. The other columns show the bands produced by the seven restriction endonucleases, before and after C-banding. The letters (a-g) represent the C-bands according to their order of appearance in each chromosome.  Arrowheads indicate C-positive heterochromatin bands only revealed by previous RE treatment. Asterisks indicate extra C-bands produced by the splitting of a control C- band after endonuclease digestion+C-banding.

**Figure 3:**
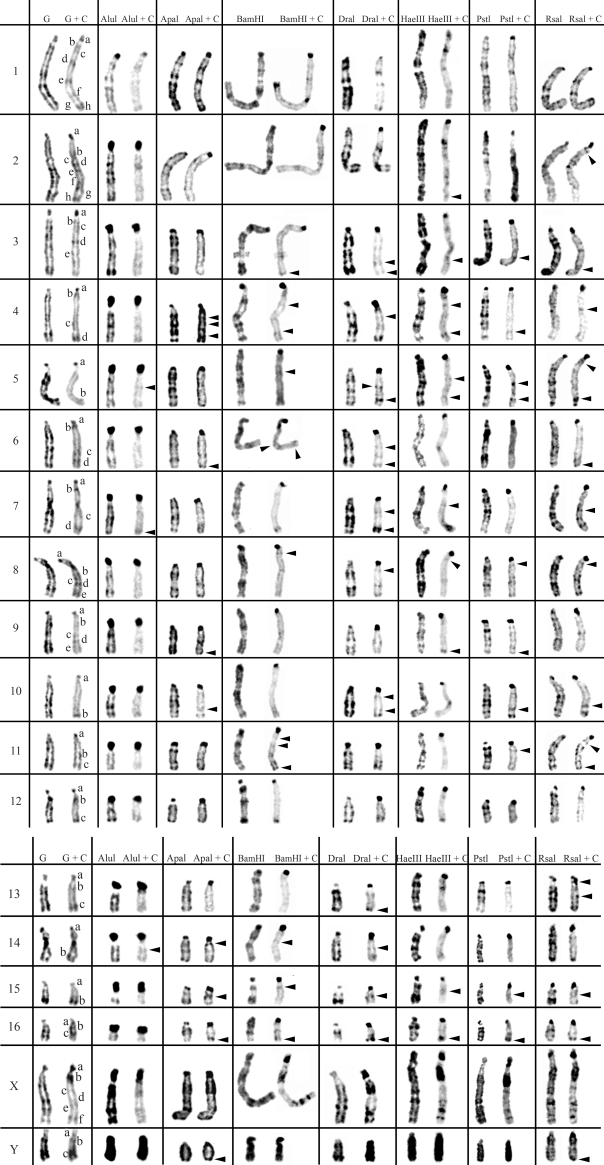
Table resume of *in situ* restriction endonuclease digestion of *Praomys tullbergi* chromosomes (2n = 34) and sequential C-banding. Control G- and C-banding of *Praomys tullbergi* chromosomes are shown on the left column. The other columns show the bands produced by the seven different restriction endonucleases, before and after C-banding. The letters (a-h) represent the C-bands according to their order of appearance in each chromosome.  Arrowheads indicate C-positive heterochromatin bands only revealed by previous RE treatment.

## Materials and Methods

### Chromosome preparations

The material analyzed consisted of chromosomal preparations of *Cricetus cricetus* (CCR), *Peromyscus eremicus* (PER) and *Praomys tullbergi* (PTU), prepared from fibroblast cell lines obtained from the cell and tissue collection maintained at the Department of Systematics and Evolution, Muséum National d'Histoire Naturelle (MNHN), Paris. Standard cell culture was followed as described elsewhere (Chaves *et al.*, 2004a) in order to prepare fixed chromosome spreads.

### GTD-banding

Air dried slides were aged at 65 °C for 5 h or overnight and then subjected to standard G-banding procedures with trypsin (Seabright, 1971). The same slides were subsequently fixed with formaldehyde and used for C-banding (Chaves *et al.*, 2002). Slides were stained with DAPI for a better contrast. The inversion of the DAPI color in Adobe Photoshop (version 7.0) revealed the chromosomes G-banding pattern (GTD-banding, G-bands by trypsin with DAPI).

### *In situ* RE digestion

Air dried slides were aged at 65 °C for 6 h and then submitted to *in situ* restriction enzyme (RE) digestion. The seven restriction enzymes used (AluI, ApaI, BamHI, DraI, HaeIII, PstI and RsaI) were diluted in buffers indicated by the manufacturer (Invitrogen Life Technologies) to give final concentrations of 30U per 100 μL of solution. One hundred microliters of the desired solution was placed on slides that were then covered with coverslips and incubated in a moist chamber for 16 h at 37 °C. Control slides were incubated only with buffer under the same conditions. Prior to C-banding, the slides were fixed with formaldehyde. Finally, the slides were stained with DAPI (the inversion of the DAPI color revealed the RE-banding). The residual bands obtained after the endonuclease digestion were suitable for chromosome identification and karyotype organization.

### CBP-banding sequential to G-bands or RE-bands

The C-banding technique was performed sequentially to G-bands or to RE banding and was carried out after distaining the slides. CBP-banding (C-bands by barium hydroxide using propidium iodide) was done using the standard procedure of Sumner (1972), but with propidium iodide as counterstain. The results presented below are representative of at least 35 metaphases from at least five independent experiments done for each endonuclease.

### Chromosome observation

Chromosomes were observed with a Zeiss Axioplan 2 imaging microscope coupled to an Axiocam digital camera with AxioVision software (version Rel. 4.5). Digitized photos were prepared for printing in Adobe Photoshop (version 7.0); contrast and color optimization were the functions used and affected the whole of the image equally.

## Results and Discussions

The karyotype of *Cricetus cricetus* has 22 chromosomes, being the first description performed by Matthey (1952). This karyotype is composed by five meta/submetacentric chromosome pairs, four submetacentric and one acrocentric, being the X chromosome a large meta/submetacentric. The karyotype of *Peromyscus eremicus* has 48 chromosomes, in agreement with the initial description by Hsu and Arrighi (1966). In this species, all of the chromosomes are submetacentric, being the X chromosome a large submetacentric and the Y a small submetacentric. The karyotype of *Praomys tullbergi* has 34 chromosomes in which all of the autosomes are acrocentric, the Y chromosome is a small acrocentric and the X chromosome is a large submetacentric (Matthey, 1958; Qumsiyeh *et al.*, 1990; Capanna *et al.*, 1996; Meles *et al.*, 2008). The first description of this karyotype was reported by Matthey (1958).

The action of all seven different REs and REs+C-banding on *Cricetuscricetus*, *Peromyscus eremicus* and *Praomys tullbergi* chromosomes are presented in Figures 1,2 3 and respectively. The residual bands seen after digestion with endonucleases AluI, ApaI, BamHI, DraI, HaeIII, PstI and RsaI (left column for each enzyme shown in Figure 1-3) are mainly G-like and suitable for chromosome identification. Although each restriction endonuclease was expected to yield a specific banding pattern, in practice most of the banding patterns overlapped. Nevertheless some endonucleases (*e.g.* ApaI, PstI and RsaI in chromosomes of *Cricetus cricetus*, BamHI, PstI and RsaI in *Peromyscus eremicus* and HaeIII, PstI and RsaI in *Praomys tullbergi*) produced a higher banding contrast. AluI was, perhaps, the used enzyme that produced the smallest number of bands but the higher contrast banding pattern. It is important to refer that the banding patterns produced by each RE are reproducible and can be used in sequential experiment procedures without loss of chromosome morphology (Chaves *et al.*, 2002; Adega *et al.*, 2005).

In a general overview, the C-positive heterochromatin (Figure 1-3, right chromosome in each column, showing control C-banding and RE+C-banding) is mainly found at the centromeres of most chromosomes, although some C-bands can also be seen at interstitial and telomeric locations. In the individuals analyzed, some heterochromatin polymorphism of minor significance were detected, *i.e.*, variation in the banding patterns of homologous chromosomes of the same pair, as also reported for pig (Adega *et al.*, 2005) and some Tayassuidae species (Adega *et al.*, 2007) chromosomes. The heterochromatin polymorphisms detected in the chromosomes of the studied species were not considered for the analysis relatively to the characterization of CH here presented, because they might not be representative of the population.

At least three major classes of CH were identified in the species studied in this work: (peri)centromeric, interstitial and telomeric (Figure 1-3). With RE+C-banding treatment, these major C-positive heterochromatin blocks could be discriminated in at least 26 C-positive heterochromatin subclasses in the autosomal complement of *Cricetus cricetus* [seven in (peri)centromeric regions, 13 in interstitial regions and six in telomeric regions] and three C-positive heterochromatin subclasses in the CCRX chromosome [one (peri)centromeric and two in interstitial regions] (cf. Figure 1). In *Peromyscus eremicus* chromosome*s* (Figure 2), the RE+C-banding treatment discriminated at least 26 C-positive heterochromatin subclasses in the autosomal complement [seven in (peri)centromeric regions, 13 in interstitial regions and six in telomeric regions], three C-positive heterochromatin subclasses in the PERX chromosome (one in the centromeric region and two in interstitial regions) and two in the PERY chromosome (one centromeric and one subtelomeric). Finally, in *Praomys tullbergi*, the RE+C-banding treatment (Figure 3) discriminated the major C-positive heterochromatin blocks into at least 45 C-positive heterochromatin subclasses in the autosomal complement (two in centromeric regions, 35 in interstitial regions and eight in telomeric regions), four C-positive heterochromatin subclasses in the PTUX chromosome (one in the centromeric region and three in interstitial regions) and three in the PTUY chromosome (one in the centromeric region and two in interstitial regions).

### Constitutive Heterochromatin (C-positive heterochromatin) characterization in *Cricetus cricetus*

Control experiment (G+C-banding) show that all the chromosomes of *Cricetus cricetus* exhibit large (peri)centromeric C-bands that in most cases consist of two blocks of CH (exception goes to CCR7, CCR8 and CCR10 chromosomes which show only one block of CH). Notice the very large centromeric CH block of the only acrocentric chromosome of the karyotype, CCR7. All the chromosomes except CCR3, CCR8 and CCR10 exhibit interstitial C-positive heterochromatin. Telomeric C-bands can be seen on chromosomes CCR1, CCR3, CCR5, CCR6, and CCR9.

Incubation of this species chromosomes with restriction endonucleases followed by C-banding revealed C-bands heterogeneity (Figure 1), being verified that (peri)centromeric, interstitial or telomeric C-bands present a different molecular nature, exhibiting different restriction patterns when submitted to the same panel of REs. This is not surprising as similar results have been reported for other species (Babu, 1988; Fernández-García *et al.*, 1998; Chaves *et al.*, 2004b; Adega *et al.*, 2005, 2007).

The arrowheads in Figure 1 indicate C-bands revealed only after RE treatment (cryptic C-bands). Of the endonucleases used here, BamHI+C-banding was the one that produced the most evident effect in CH sequences of the *Cricetus cricetus* chromosomes. See for instance chromosomes CCR7, CCR8, CCR9 and CCR10, being observed less intense bands in comparison with the control chromosomes. This enzyme, along with ApaI+C-banding and RsaI+C-banding, produced the partition of the (peri)centromeric CH band at chromosomes CCR7 and CCR10 into two distinct CH blocks, thus revealing the occurrence of two instead of one (peri)centromeric CH block [bands identified with an asterisk in Figure 1]. Some enzymes seem to have a drastic effect resulting in a more accentuated contrast pattern in the (peri)centromeric regions of some chromosomes. See, for example, chromosomes CCR1 and CCR6 with DraI+C-banding, CCR5 with BamHI+C-banding, CCR9 with BamHI+C-banding and DraI+C-banding.

### Constitutive Heterochromatin (C-positive heterochromatin) characterization in *Peromyscus eremicus*

In the control experiment (G+C-banding) the majority of *Peromyscus eremicus* chromosomes exhibit large (peri)centromeric C-bands (Figure 2, left column), and in some of these chromosomes, the C-banding spreads from the centromeric region to the p arm telomere, apparently covering all the p arm, *e.g.*, chromosomes PER9 and PER17. In some chromosomes, this band seems to be split in two C-bands, one clearly centromeric and the other covering the chromosome p arm (chromosomes PER2, PER3 and PER4). Chromosomes PER11 and PER16 display two well-defined bands of (peri)centromeric CH, although this may have been an artifact caused by the small size of the p arms. Chromosomes PER1 and PERY apparently display the lowest amount of heterochromatin in control G+C-banding, showing PER1 only a small centromeric CH band. The situation observed in the PERY is not usual for most of the mammals' species, once this chromosome is usually the more heterochromatic of the whole complement. Some of the chromosomes exhibit C-bands at interstitial locations, presenting chromosome PERX the highest number of these bands (at least six). Telomeric C-bands can be observed in some chromosomes of this species, *e.g.*, PER6, PER11, PER12, PER15 and PER16 (Figure 2).

When C-banding was applied after *in situ* REs digestion to the chromosomes of this species, it was possible to verify that its CH shows some degrees of heterogeneity (Figure 2). The arrowheads in Figure 2 indicate C-bands revealed only after treatment with endonucleases (cryptic C-bands). From the REs used in this work, RsaI+C-banding, PstI+C-banding and BamHI+C-banding, were the enzymes that revealed the greatest number of CH bands not previously detected by the control G+C-banding.

In a general analysis, AluI was the enzyme that produced the most divergent effects on the CH of *Peromyscus eremicus* chromosomes. In some cases, such as in chromosomes PER1 and PER6, some C-bands seem to have undergone a greater reduction or even have, apparently disappeared when compared with control experiment, while in other cases, such as chromosomes PER7 and PER16, the CH was apparently unaffected by treatment with this enzyme.

The p arms CH of PER2, PER3 and PER4 chromosomes are particularly interesting in what respects to its molecular nature. In these heterochromatic arms the CH reveals a high heterogeneity, what is verified by the different restriction patterns produced by the enzymes at these CH regions. For instance in the p arm of PER 2 there were recognized two C-bands in the control G+C-banding; after AluI+C restriction a lesser intensity of one of these bands was observed and ApaI+C-banding and HaeIII+C-banding seem to reveal an extra C-band, by splitting one of the previous in two [bands evidenced with an asterisk (*) in Figure 2].

### Constitutive Heterochromatin (C-positive heterochromatin) characterization in *Praomys tullbergi*

From the studied species, *Praomys tullbergi* (Figure 3) is the one whose chromosomes exhibit the lower amount of centromeric CH in the control experiment (G+C-banding). In some chromosomes, centromeric CH is almost as abundant as interstitial CH, in contrast to the observed for the majority of the chromosomes from the other species here analyzed. However, the chromosomes PTU5 and PTU10 in the control experiment, present a small centromeric CH band and apparently do not reveal interstitial bands. The majority of the chromosomes display several interstitial CH bands, presenting the chromosomes PTU1 and PTU2 the greatest number of these bands. Telomeric C-bands are clearly distinguishable in some chromosomes, *e.g.*, chromosomes PTU10, PTU12 and PTU15. The PTUX chromosome presents three distinct classes of CH, centromeric, interstitial and telomeric. PTUY chromosome exhibits a centromeric band and two interstitial C-bands.

When C-banding was applied after *in situ* REs digestion to the chromosomes of this species, it was possible to verify that its CH shows some degrees of heterogeneity, just as it was described for the other two rodent species studied in this work. AluI+C-banding produced the higher contrast between the centromeric *versus* interstitial/telomeric CH classes; digestion with AluI greatly decreased the interstitial/telomeric CH while, simultaneously, evidenced the centromeric heterochromatin. See for instance, chromosomes PTU15 or PTU16, whose centromeres showed in the control G+C-banding an almost absence of CH, and after the AluI+C-banding the centromeres showed large centromeric CH blocks. Digestion with DraI seems to highlight the telomeric CH after C-banding, *e.g.*, chromosomes PTU7 and PTU16. RsaI+C-banding seems to produce the most similar results with the control G+C-banding, however also discriminating cryptic C-bands, such as the ones observed in chromosomes PTU4, PTU5, PTU10 or PTUX. Other endonucleases also disclosed cryptic C-bands, especially DraI+C-banding, BamHI+C-banding or HaeIII+C-banding.

These special bands are very interesting from the CH molecular nature point of view, since their disclosure is probably dependent on sequence modifications (not yet clearly understood) induced by the REs, leading for instance, to an increase of the stain capacity to bind a specific chromosome region (Gosálvez *et al.*, 1997; Nieddu *et al.*, 1999; Chaves *et al.*, 2004b). Whatever the mechanism behind these sequences modification, RE digestion triggers it, revealing “hidden” C-bands. Curiously, and from several different works in different species, these sequences not detected by classical C-banding have proven to correspond to clinical (*Sus scrofa*, Adega *et al.*, 2005) or evolutionary breakpoints (Tayassuidae, Adega *et al.*, 2007).

### Inter-species constitutive heterochromatin (C-positive heterochromatin)

A general comparison of the amount, distribution and molecular nature of C-positive heterochromatin in the three Rodentia species, suggests that the CH of these karyotypes is extremely different. Evidence comes from the detailed combined analysis of the different REs+C-banding patterns disclosed on the karyotypes of these species. The application of a seven REs panel to the chromosomes of three different rodent species, *Cricetus cricetus*, *Peromyscus eremicus* (Cricetidae) and *Praomys tullbergi* (Muridae), allowed a characterization of its CH and the recognition of its molecular heterogeneity. These results are a clear reflex of the different C-positive heterochromatin composition of these karyotypes, possible to observe by the different REs actions on the respective chromosome's bands.

*Cricetus cricetus* has an almost entirely meta/submetacentric karyotype (with only one acrocentric pair), with the CH primarily located in (peri)centromeric regions. Most of the chromosomes in this species exhibit two very large blocks at (peri)centromeric location, which suggested the occurrence of dicentric Robertsonian translocations or, alternatively, heterochromatin additions during the course of this karyotype evolution. The other Cricetidae species, *Peromyscus eremicus,* has a very distinct karyotype that comprises only submetacentric chromosomes. This karyotype also displays great amounts of CH, especially located at the (peri)centromeric regions, being the p arms of some chromosomes composed entirely by this repetitive component of the genome. The heterochromatin of p arms revealed a great heterogeneity, what implies a different molecular composition, which is certainly indicative of the coexistence of different satellite DNA families or variants at these chromosome regions.

The species *Praomys tullbergi*, with a complete acrocentric autosome complement, it is the one whose chromosomes exhibit the lower amount of centromeric CH in the control experiment (G+C-banding), and in some cases, interstitial heterochromatin is almost as abundant as centromeric heterochromatin. This uniform and scattered distribution, together with the higher number of CH subclasses identified in *Praomys tullbergi* chromosomes (52 subclasses) suggests that this species has a more derivative karyotype than the other two genomes analyzed, probably originated by a great number of complex chromosomal rearrangements. This is based on the assumption that heterochromatic rich regions act as hotspots for the occurrence of chromosome rearrangements (Yunis and Yasmineh, 1971; Peacock *et al.*, 1982, John, 1988; Chaves *et al.*, 2004b), either by promoting the chromosome structural rearrangements that reshape karyotypes or by being fragile regions prone to chromosome breakage, and consequently to chromosome rearrangement, representing remnants of these events. The suggestion that the karyotype of *Praomys tullbergi* was originated by the occurrence of a high number of complex chromosomal rearrangements is supported by the work of Meles *et al.* (2008), where it was detected telomeric interstitial sequences in several chromosome arms of this species, probably the result of tandem fusions.

Finally, it is worth mentioning the value of *in situ* RE digestion with sequential C-banding as an alternative tool for the study of Rodentia chromosomes CH, especially when other techniques are not available, as fluorescent *in situ* hybridization with different repetitive sequences.

## References

[Adegaetal2005] Adega F., Chaves R., Guedes-Pinto H. (2005). Chromosome restriction enzyme digestion in domestic pig (*Sus scrofa*). Constitutive heterochromatin arrangement. Genes Genet Syst.

[Adegaetal2007] Adega F., Chaves R., Guedes-Pinto H. (2007). Constitutive heterochromatin characterization of white-lipped and collared peccaries (Tayassuidae). J Genet.

[ArrighiandHsu1971] Arrighi F.E., Hsu T.C. (1971). Localization of heterochromatin in human chromosomes. Cytogenetics.

[Babu1988] Babu A., Verma R.S. (1988). Heterogeneity of heterochromatin of human chromosomes as demonstrated by restriction endonuclease treatment. Heterochromatin: Molecular and Structural Aspects.

[BauchanandHossain1999] Bauchan G.R., Hossain M.A. (1999). Constitutive heterochromatin DNA polymorphisms in diploid *Medicago sativa* ssp. *falcata*. Genome.

[Capannaetal1996] Capanna E., Codjia J.T.C., Chrysostome C., Civitelli M.V. (1996). Les chromosomes des rongeurs du Benin (Afrique de l'Ouest): 3 Murinae. Rend Fis Acc Lincei.

[Chavesetal2000] Chaves R., Guedes-Pinto H., Heslop-Harrison J., Schwarzacher T. (2000). The species and chromosomal distribution of the centromeric alpha-satellite I sequence from sheep in the tribe Caprini and other Bovidae. Cytogenet Cell Genet.

[Chavesetal2002] Chaves R., Adega F., Santos S., Guedes-Pinto H., Heslop-Harrinson J.S. (2002). *In situ* hybridization and chromosome banding in mammalian species. Cytogenet Genome Res.

[Chavesetal2004a] Chaves R., Frönicke L., Guedes-Pinto H., Wienberg J. (2004a). Multidirectional chromosome painting between the Hirola antelope (*Damaliscus hunteri*, Alcelaphini, Bovidae), sheep and human. Chromosome Res.

[Chavesetal2004b] Chaves R., Santos S., Guedes-Pinto H. (2004b). Comparative analysis (Hippotragini *versus* Caprini, Bovidae) of X-chromosome's constitutive heterochromatin by *in situ* restriction endonuclease digestion: X-chromosome constitutive heterochromatin evolution. Genetica.

[Comings1973] Comings D.E., Casperson T., Zeck L. (1973). Biochemical mechanisms of chromosome banding and color banding with acridine orange. Chromosome Identification - Techniques and Applications in Biology and Medicine.

[Corradinietal2007] Corradini N., Rossi F., Giordano E., Caizzi R., Vern F., Dimitri P. (2007). *Drosophila melanogaster* as a model for studying protein-encoding genes that are resident in constitutive heterochromatin. Heredity.

[Dimitrietal2004] Dimitri P., Corradini N., Rossi F., Verní F. (2004). The paradox of functional heterochromatin. Bioessays.

[Dimitrietal2005] Dimitri P., Verní F., Mei E., Rossi F., Corradini N. (2005). Transposable elements as artisans of the heterochromatic genome. Cytogenet Genome Res.

[Fernandez-Garciaetal1998] Fernández-García J.L., Martínez-Trancón M., Rabasco A., Padilla J.A. (1998). Characterization of the heterochromatic chromosome regions in sheep. Genes Genet Syst.

[Gallenietal1992] Galleni L., Stanyon R., Tellini A., Giordano G., Santini L. (1992). Karyology of the Savi pine vole, *Microtus savii* (De Sélys-Longchamps, 1838) (Rodentia, Arvicolidae): G-, C-, DA/DAPI, and AluI-bands. Cytogenet Cell Genet.

[Garciaetal2000a] García L., Ponsá M., Egozcue J., García M. (2000a). Comparative chromosomal analysis and phylogeny in four *Ctenomys* species (Rodentia, Octodontidae). Biol J Linn Soc.

[Garciaetal2000b] García L., Ponsá M., Egozcue J., García M. (2000b). Cytogenetic variation in *Ctenomys perrensi* (Rodentia, Octodontidae). Biol J Linn Soc.

[Gosalvezetal1997] Gosálvez J., López-Fernández C., Goyanes R., Mezzanotte V., Henriques-Gil N., Parker J.S., Puertas M.J. (1997). Chromosome differentiation using nucleases: An overview. Chromosomes Today.

[HolmquistandDancis1979] Holmquist G.P., Dancis B. (1979). Telomere replication, kinetochore organizers, and satellite DNA evolution. Proc Natl Acad Sci USA.

[HsuandArrighi1966] Hsu T.C., Arrighi F.E. (1966). Chromosomal evolution in the genus *Peromyscus* (Cricetidae, Rodentia). Cytogenetics.

[Jobseetal1995] Jobse C., Buntjer J.B., Haagsma N., Breukelman H.J., Beintema J.J., Lenstra J.A. (1995). Evolution and recombination of bovine DNA repeats. J Mol Evol.

[John1988] John B., Verma R.S. (1988). The biology of heterochromatin. Heterochromatin: Molecular and Structural Aspects.

[Johnetal1985] John B., King M., Schweizer D., Mendelak M. (1985). Equilocality of heterochromatin distribution and heterochromatin heterogeneity in acridid grasshoppers. Chromosoma.

[Leeetal1999] Lee C., Stanyon R., Lin C.C., Ferguson-Smith M.A. (1999). Conservation of human gamma-X centromeric satellite DNA among primates with an autosomal localization in certain Old World monkeys. Chromosome Res.

[Matthey1952] Matthey R. (1952). Chromosomes des Muridae (Microtinae et Cricetinae). Chromosoma.

[Matthey1958] Matthey R. (1958). Les chromosomes et la position systématique de quelques Murinae africains (Mammalia-Rodentia). Acta Trop.

[Melesetal2008] Meles S., Adega F., Guedes-Pinto H., Chaves R. (2008). The karyotype of *Praomys tullbergi* (Muridae, Rodentia): A detailed characterization. Micron.

[Niedduetal1999] Nieddu M., Rossino R., Pichiri G., Rocchi M., Setzu M.D., Mezzanotte R. (1999). The efficiency of *in situ* hybridization on human chromosomes with alphoid DNAs is enhanced by previous digestion with AluI and TaqI. Chromosome Res.

[PathakandArrighi1973] Pathak S., Arrighi F.E. (1973). Loss of DNA following C-banding procedures. Cytogenet Cell Genet.

[Peacocketal1982] Peacock W.J., Dennis E.S., Gerlach W.L. (1982). DNA sequence changes and speciation. Prog Clin Biol Res.

[Pieczarkaetal1998] Pieczarka J.C., Nagamachi C.Y., Muniz J.A.P.C., Barros R.M.S., Mattevi M.S. (1998). Analysis of constitutive heterochromatin of *Aotus* (Cebidae, Primates) by restriction enzyme and fluorochrome bands. Chromosome Res.

[ProbstandAlmouzni2008] Probst A.V., Almouzni G. (2008). Pericentric heterochromatin: Dynamic organization during early development in mammals. Differentiation.

[Qumsiyehetal1990] Qumsiyeh M.B., King S.W., Arroyo-Cabrales J., Aggundey I.R., Schlitter D.A., Baker R.J., Morrow K.J. (1990). Chromosomal and protein evolution in morphologically similar species of *Praomys* sensu lato (Rodentia, Muridae). *J Hered*.

[Roccoetal2002] Rocco L., Morescalchi M.A., Costagliola D., Stingo V. (2002). Karyotype and genome characterization in four cartilaginous fishes. Gene.

[Rossietal2007] Rossi F., Moschetti R., Caizzi R., Corradini N., Dimitri P. (2007). Cytogenetic and molecular characterization of heterochromatin gene models in *Drosophila melanogaster*. Genetics.

[Saitoetal2007] Saito Y., Edpalina R.R., Abe S. (2007). Isolation and characterization of salmonid telomeric and centromeric satellite DNA sequences. Genetica.

[Santosetal2004] Santos S., Chaves R., Guedes-Pinto H. (2004). Chromosomal localization of the major satellite DNA family (FA-SAT) in the domestic cat. Cytogenet Genome Res.

[Schmidetal2002] Schmid M., Haaf T., Steinlein C., Nanda I., Mahony M. (2002). Chromosome banding in Amphibia: XXV. Karyotype evolution and heterochromatin characterization in Australian *Mixophyes* (Anura, Myobatrachidae). Cytogenet Genome Res.

[Seabright1971] Seabright M. (1971). A rapid banding technique for human chromosomes. Lancet.

[Shore2001] Shore D. (2001). Telomeric chromatin: Replicating and wrapping up chromosome ends. Curr Opin Genet Dev.

[Singer1982] Singer M.F. (1982). Highly repeated sequences in mammalian genomes. Int Rev Cytol.

[SlamovitsandRossi2002] Slamovits C.H., Rossi M.S. (2002). Satellite DNA: Agent of chromosomal evolution in mammals. A review. J Neotrop Mammal.

[Sumner1972] Sumner A.T. (1972). A simple technique for demonstrating centromeric heterochromatin. Exp Cell Res.

[VanDenBusscheetal1993] Van Den Bussche R.A., Honeycutt R.L., Baker R.J. (1993). Restriction endonuclease digestion patterns of harvest mice (*Reithrodontomys*) chromosomes: A comparison to G-bands, C-bands, and *in situ* hybridization. Genetica.

[VermaandBabu1995] Verma R.S., Babu A. (1995). Human Chromosomes – Principles and Techniques.

[WayeandWillard1989] Waye J.S., Willard H.F. (1989). Concerted evolution of alpha satellite DNA: Evidence for species specificity and a general lack of sequence conservation among alphoid sequences of higher primates. Chromosoma.

[Wichmanetal1991] Wichman H.A., Payne C.T., Ryder O.A., Hamilton M.J., Maltbie M., Baker R.J. (1991). Genomic distribution of heterochromatic sequences in equids: Implications to rapid chromosomal evolution. J Hered.

[YunisandYasmineh1971] Yunis J.J., Yasmineh W.G. (1971). Heterochromatin, satellite DNA, and cell function. Structural DNA of eukaryotes may support and protect genes and aid in speciation. Science.

